# Disease specific characteristics of fetal epigenetic markers for non-invasive prenatal testing of trisomy 21

**DOI:** 10.1186/1755-8794-7-1

**Published:** 2014-01-08

**Authors:** Ji Hyae Lim, Da Eun Lee, So Yeon Park, Do Jin Kim, Hyun Kyong Ahn, You Jung Han, Moon Young Kim, Hyun Mee Ryu

**Affiliations:** 1 Laboratory of Medical Genetics, Medical Research Institute, Cheil General Hospital and Women’s Healthcare Center, Seoul, Korea; 2 Department of Obstetrics and Gynecology, Cheil General Hospital and Women’s Healthcare Center, Kwandong University College of Medicine, Seoul, Korea

**Keywords:** Trisomy 21, Non-invasive prenatal testing, Epigenetic markers

## Abstract

**Background:**

Non-invasive prenatal testing of trisomy 21 (T21) is being actively investigated using fetal-specific epigenetic markers (EPs) that are present in maternal plasma. Recently, 12 EPs on chromosome 21 were identified based on tissue-specific epigenetic characteristics between placenta and blood, and demonstrated excellent clinical performance in the non-invasive detection of fetal T21. However, the disease-specific epigenetic characteristics of the EPs have not been established. Therefore, we validated the disease-specific epigenetic characteristics of these EPs for use in non-invasive detection of fetal T21.

**Methods:**

We performed a high-resolution tiling array analysis of human chromosome 21 using a methyl-CpG binding domain-based protein (MBD) method with whole blood samples from non-pregnant normal women, whole blood samples from pregnant normal women, placenta samples of normal fetuses, and placenta samples of T21 fetuses. Tiling array results were validated by bisulfite direct sequencing and qPCR.

**Results:**

Among 12 EPs, only four EPs were confirmed to be hypermethylated in normal placenta and hypomethylated in blood. One of these four showed a severe discrepancy in the methylation patterns of T21 placenta samples, and another was located within a region of copy number variations. Thus, two EPs were confirmed to be potential fetal-specific markers based on their disease-specific epigenetic characteristics. The array results of these EPs were consisted with the results obtained by bisulfite direct sequencing and qPCR. Moreover, the two EPs were detected in maternal plasma.

**Conclusions:**

We validated that two EPs have the potential to be fetal-specific EPs which is consistent with their disease-specific epigenetic characteristics. The findings of this study suggest that disease-specific epigenetic characteristics should be considered in the development of fetal-specific EPs for non-invasive prenatal testing of T21.

## Background

Prenatal testing is an integral component of modern obstetric practice and is commonly performed in professional medical organizations worldwide. The primary aim of prenatal testing is the diagnosis of fetal aneuploidies, such as trisomy 21 (T21, Down syndrome), trisomy 18 (Edwards syndrome) and trisomy 13 (Patau syndrome) [1]. Although the majority of fetuses with aneuploidy result in spontaneous termination during fetal development, T21 has the highest survival rate, affecting 1 in 800 births [2]. Therefore, prenatal detection of T21 is considered the most common and important aspect of prenatal genetic testing. Current prenatal screening tests of T21 have greatly improved by using a combination of maternal serum markers and fetal sonographic markers such as nuchal translucency [3-6]. However, positive screening results require confirmation with diagnostic testing, such as amniocentesis or chorionic villus sampling (CVS) [7]. However, both sampling procedures are invasive, and are associated with significant risks to the fetus and mother, including the potential loss of a healthy fetus [7,8]. For this reason, invasive prenatal diagnostic tests are currently preformed only in high-risk pregnancies or in pregnancies with increased maternal age and/or family history of having a child with an inherited disease. Therefore, developing a reliable method for non-invasive prenatal testing (NIPT) for fetal T21 is of critical importance in prenatal care.

To perform NIPT, a source of fetal genetic material that could be sampled without harm to the fetus is needed. In 1997, Lo et al. discovered the existence of cell-free fetal DNA (cff-DNA) in maternal circulation [9]. It is rapidly cleared from maternal blood within two hours of delivery and constitutes approximately 10% of the total DNA in maternal plasma [10,11]. Moreover, it has recently been found that the entire fetal genome, in the form of cff-DNA, is present in maternal blood [12]. Therefore, cff-DNA is regarded as a promising new material for the development of reliable NIPT of fetal T21.

Various methods have been applied for the NIPT of fetal T21 using cff-DNA. Recently, the NIPT for T21 based on next-generation sequencing reach sensitivities and specificities of over 99% and have already been applied in the clinical setting [13-15]. However, next-generation sequencing technologies are of high cost and not easily accessible to diagnostic laboratories, because requires major and expensive infrastructure. To overcome these issues, epigenetic modifications as fetal-specific signatures to detect cff-DNA from circulating maternal DNA have been investigated. A number of fetal-specific epigenetic markers (EPs) on chromosome 21 that could be used for the NIPT of fetal T21 have been reported. However, the diagnostic accuracy of these fetal-specific EPs for the NIPT of fetal T21 range from 80 to 100%, depending on the markers and methods used [16-18].

In a previous study, Papageorgiou *et al*. identified 12 tissue-specific differentially methylated regions (DMRs) that are hypermethylated in placental DNA of the euploid fetus and hypomethylated in non-pregnant female normal whole blood DNA using methylated DNA immunoprecipitation and tiling microarray [19]. The DMRs were suggested for use as fetal-specific EPs in the NIPT of fetal T21. They proposed a fetal-specific DNA methylation ratio method using the EPs for fetal T21 detection and reported excellent clinical performance (both the sensitivity and specificity were 100%) [18]. However, the EPs were selected based on the only tissue-specific epigenetic characteristics between placenta and blood and their disease-specific epigenetic characteristics have not been validated.

The aim of this study was to validate disease-specific epigenetic characteristics of EPs for the NIPT of fetal T21 in whole blood from non-pregnant euploid women, whole blood from pregnant euploid women, euploid fetal placenta, and T21 fetal placenta using a high-resolution tiling array analysis of human chromosome 21 and to select the effective EPs that have the potential to be used as fetal-specific EPs for the NIPT of fetal T21.

## Methods

### Ethics statement

This study was conducted according to the principles expressed in the Declaration of Helsinki. Appropriate institutional review board approval for this study was obtained from the Ethics Committee at Cheil General Hospital (#CGH-IRB-2011-85). All patients provided written informed consent for the collection of samples and subsequent analysis.

### Sample processing

Pregnant women with euploid or T21 fetuses and non-pregnant euploid women who attended the Department of Obstetrics and Gynecology at Cheil General Hospital, Korea were recruited between March and December 2011. Maternal peripheral blood samples were collected into tubes containing EDTA just before obstetric procedures (such as CVS) in the first trimester. All placenta samples were obtained at CVS. Maternal peripheral blood samples were centrifuged at 1,600 g for 10 min. The peripheral blood cell portion was re-centrifuged at 2,500 g for 10 min, and any residual plasma was removed. DNA was extracted from maternal peripheral blood cells using the QIAamp Blood kit (Qiagen, West Sussex, UK) and from placental tissues using the QIAamp Tissue Kit (Qiagen).

### Array hybridizations

Whole blood samples from non-pregnant euploid women, whole blood samples from pregnant euploid women, euploid fetal placenta samples, and T21 fetal placenta samples from pregnant euploid women were used. Each DNA sample was extracted from placental tissues and maternal blood cells, and then sonicated and subjected to a methyl-CpG binding domain-based protein (MBD) method. The subsequent product was amplified and labeled using a Genomic DNA Enzymatic Labeling Kit (Agilent Technologies, DE, USA) and hybridized to a customized chip specific for chromosome 21. The array platforms were composed of 0.4 million 50-mer to 60-mer oligonucleotides covering chromosome 21 at a median probe density of 1 per 60 bp. Microarray protocols, including labeling, hybridization and post-hybridization washing procedures, were performed as recommended by the manufacturer and are available at https://www.home.agilent.com. Briefly, the labeled MBD (Cyanine 5-dUTP: Cy5) DNA and reference DNA (Cyanine 3-dUTP: Cy3) were cleaned using Amicon filters (Millipore Corporation, MA, USA). Competitive hybridization onto the microarray was then performed using a Methylation Hybridization Kit (Agilent Technologies) in a rotating SureHyb chamber at 67°C for 40 h according to the manufacturer’s instructions. Washed slides were scanned using a High-Resolution C Scanner (Agilent Technologies) and fluorescence was evaluated using Feature Extraction software (Agilent Technologies).

### Data analysis of the tiling oligonucleotide arrays

The two signal values were normalized using background subtraction, and signal ratio (MBD/input), signal log ratio [log_2_(MBD/input)], P[X], and P were obtained using Agilent Genomic Workbench software (Agilent Technologies). The log_2_ value is the Cy5:Cy3 fluorescence ratio (methylated DNA recovered by MBD capture: total input DNA) for each probe, converted to a log_2_ scale, and represents a relative measure of the amount of methylated DNA at each locus. We applied median and Lowess normalization to the raw data and filtered outlier probes to remove low-quality data points. The P[X] and P values, which are used in the MBD array analysis to obtain a binding call, were defined as the probability that the X value deviates from the Gaussian distribution of X values of the entire genome of a sample. Here, the X value for a probe was the difference between the MBD and the input signals after adjusting for the symmetry of its distribution. The value for a probe was calculated as an average X, taking into account the signals of the neighboring probes (within 1 kb of the probe). Finally, we defined hypermethylation as a normalized log_2_ ratio > 0.5, hypomethylation as a normalized log_2_ ratio between 0.001 and 0.499, and unmethylation as a normalized log_2_ ratio = 0.

### Confirmation of copy number variations

The accurate quantification of fetal-specific EPs by methylation-based DNA discrimination is of critical importance in the NIPT of fetal T21 using EPs, because such fetal-specific EPs on chromosome 21 are used in the detection of fetal T21 by direct comparison with a placenta-derived DNA methylation marker on a reference chromosome. Therefore, the fetal-specific EPs should be selected from regions without copy number variations (CNVs), which can result in dosage imbalances and interfere with the correct interpretation of results. Therefore, the DNA sequence of the 12 EPs was checked for the presence of CNVs or segmental duplication by searching the Database of Genomic Variants (http://dgv.tcag.ca/dgv/app/home). The EPs that were free of CNVs and segmental duplications were selected as fetal-specific EPs with potential for the NIPT of fetal T21.

### Bisulfite direct sequencing

We confirmed the MBD array data using bisulfite direct sequencing. DNA samples (1 μg) were bisulfite-converted using an EpiTect Bisulfite Kit (Qiagen) according to the manufacturer’s instructions. The bisulfite-converted DNA was then amplified by PCR with primers for discrimination of the methylated and unmethylated CpG sites. The sequences of PCR primers are presented in Table 1. PCR reaction solutions contained 10 ng genomic DNA, 10 pM primers, 0.25 mM dNTPs, 1.5 mM MgCl_2_, 1 X buffer, and 0.25 U Taq polymerase per 20 μL of total reaction volume. PCR conditions included predenaturation at 95°C for 10 min, 35 cycles of 95°C for 30 sec, 56°C for 30 sec, 72°C for 40 sec, and final extension at 72°C for 10 min. After PCR amplification, PCR products were purified using a PCR purification kit (Bioneer, Daejeon, Korea) and sequenced using a PRISM BigDye Terminator Cycle Sequencing Kit (Applied Biosystems, Foster city, USA) according to manufacturer’s instructions. Sequencing products were analyzed using a PRISM 3100 Genetic Analyzer (Applied Biosystems), and electropherogram traces were interpreted using Genescan software version 3.7 (Applied Biosystems). Corresponding genotypes were assigned using Genotyper software version 3.7 (Applied Biosystems).

**Table 1:** Sequences of primers according to experiment

**Experiment**	**Sequences of primers**	
Bisulfite direct sequencing	EP6	Forward: 5′-GAT GCG TTA GAT TTA AGG GAG G-3′
		Reverse: 5′-CTC ACT CTC ACG AAA CCC CTC-3′
	EP7	Forward: 5′-GAG ATG TTT AGC GTT TGT GGG-3′
		Reverse: 5′-AAC TAA TTA CAT AAA ACC CAC CC-3′.
qPCR	EP6	Forward: 5′-TGA ATC AGT TCA CCG ACA GC-3′
		Reverse: 5′-GAA ACA ACC TGG CCA TTC TC-3′
	EP7	Forward: 5′-CCG TTA TAT GGA TGC CTT GG-3′
		Reverse: 5′-AAA CTG TTG GGC TGA ACT GC-3′
	EP9	Forward: 5′-GAC CCA GA CGA TAC CTG GAA-3′
		Reverse: 5′- CTG AAC AAA ACT CGG CTT C-3′

### Methylation quantification of bisulfite direct sequencing data

The methylation quantification of bisulfite direct sequencing data was performed as described previously [20]. Briefly, the methylation ratio of each CpG site was calculated as the peak height of C vs. the peak height of C plus the peak height of T for each CpG site, as shown in the computer-generated sequencing chromatogram extracted from the Chromas program (Version 2.32, Technelysium). A single C at the corresponding CpG site was considered to be 100% methylated, a single T was 100% unmethylated and overlapping C and T was partially methylated (0-100%).

### Noninvasive detection and methylation levels of EPs by qPCR

We used the PCR conditions described previously [18]. The sequences of PCR primers used are presented in Table 1. Each qPCR reaction was performed in a final volume of 20 μL with 20 ng of genomic DNA or 5 μL of methylated cell free DNA extracted from 1 ml of maternal plasma. We used SYBR Green PCR master mix (Eurogentec, Seraing, Belgium). The amplification program consisted of 3 minutes at 95°C, followed by 40 cycles of denaturation for 15 seconds at 95°C and annealing/extension for 30 seconds at 62°C. After amplification, melting curve analysis was performed by heating the reaction mixture from 65 to 95°C at a rate of 0.5°C/second.

For analysis of methylation levels of EPs, the delta (Δ) threshold cycle (Ct) value was calculated as ΔCt = Ct_input_ - Ct_MBD_. Input denotes the portion without MBD enrichment and MBD denotes the portion after immunoprecipitation. A positive ΔCt value represents a hypermethylated region with enrichment, and a negative ΔCt value represents a hypomethylated region without enrichment.

### Statistical analysis

Data are expressed as a mean ± standard deviation (S.D.). The clinical data and methylation levels in the study groups were compared using robust one-way analysis of variance (ANOVA), the post hoc Tamhane’s T2 test for multiple comparisons, and the t-test for comparisons between the two groups. In all tests, a threshold of *P* < 0.05 was set for statistical significance. Statistical analyses were performed using the Statistical Package for Social Sciences 12.0 (SPSS Inc., Chicago, USA).

## Results

### Clinical characteristics

The clinical characteristics of the study groups are shown in Table 2. In total, whole blood samples from non-pregnant euploid women (n = 6), whole blood samples and euploid fetal placenta samples from pregnant euploid women (n = 38), and whole blood samples and T21 fetal placenta samples from pregnant euploid women (n = 12) were used. At blood sampling, age and body mass index were no differences among all study groups (*P* > 0.05 for both). Maternal age, gestational age, and gender ratio of the fetuses was also not different between pregnant women carrying T21 fetuses and pregnant women carrying normal fetuses (*P* > 0.05 for all).

**Table 2:** Clinical characteristics of the study population

**Characteristics**	**Pregnant women carrying trisomy 21 fetuses (n = 12)**	**Pregnant women carrying normal fetuses (n = 38)**	**Non pregnant women (n = 6)**	***P *** **value**
Age (years)	32.9 ± 3.5	35.3 ± 4.1	29.5 ± 0.7	0.198^a^
Body mass index (kg/m^2^)	21.7 ± 2.5	21.4 ± 2.6	21.1 ± 0.3	0.859^a^
Gestational age (weeks)	11.8 ± 0.8	12.2 ± 0.6	-	0.182^b^
Gender-ratio of fetus (male: female)	4:8	14:24	-	-

Data are presented as mean ± standard deviation.

^a^one-way analysis of variance test.

^b^t-test.

### Selection of the fetal-specific EPs for the NIPT of fetal T21

**Figure 1:**
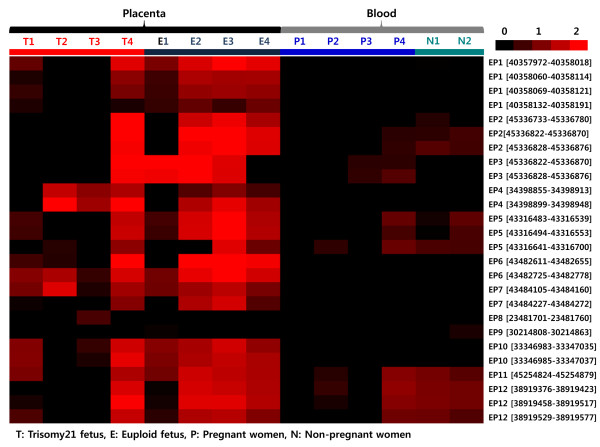
**Methylation levels of EPs in study groups using the tiling oligonucleotide arrays.** The red and black colors indicate high expression and non-expression, respectively.

In a high-resolution tiling oligonucleotide array of chromosome 21, whole blood samples from non-pregnant euploid women (n = 2), whole blood samples from pregnant euploid women (n = 4), euploid fetal placenta samples (n = 4), and T21 fetal placenta samples (n = 4) were used. Whole blood samples from pregnant euploid women and euploid fetal placental samples were paired. The array data from the 12 EPs in each sample are presented in Figure 1 and their methylation levels are given in Table 3. Among the 12 EPs, ten EPs (EP1-EP7 and EP10-EP12) were identified in probe position of our array and the other two EPs (EP8 and EP9) were located in regions vary near the probe position. Among the confirmed 12 EPs, four EPs (EP2, EP5, EP11, and EP12) were completely methylated in whole blood samples from non-pregnant women, whereas two EPs (EP3 and EP4) were completely unmethylated in one of four euploid placenta samples (Figure 1). Moreover, these EPs showed various methylation discrepancies among the T21 placenta samples that were tested. EP8 and EP9 showed a hypomethylation pattern as a whole, regardless of tissue types and the presence or absence of disease (Figure 1 and Table 3). Thus, only four EPs (EP1, EP6, EP7, and EP10) were confirmed to be hypermethylated in placental samples and hypomethylated in peripheral blood, regardless of pregnancy status (Table 3). However, EP1 was completely unmethylated in two cases among the T21 placenta samples (Figure 1). Moreover, the methylation levels of EP1 in probe regions showed significant differences between T21 placenta samples and normal placenta samples (Table 3).

**Table 3:** Methylation levels of EPs according to tissue type

**EP marker**	**Probe position**	**Placenta (Log**_**2**_**)**		**Normal blood (Log**_**2**_**)**		***P *** **value**
	**(Start-Stop)**	**Trisomy 21**	**Normal**	**Pregnant**	**Non-pregnant**	
EP1	40357972-40358018	0.954 ± 1.087	2.495 ± 0.662^d,e^	0.000 ± 0.000	0.037 ± 0.037	0.006
	40358060-40358114	0.501 ± 0.678	1.663 ± 0.533^d,e^	0.000 ± 0.000	0.000 ± 0.000	0.006
	40358069-40358121	0.513 ± 0.581	1.496 ± 0.468^d,e^	0.000 ± 0.000	0.000 ± 0.000	0.005
	40358132-40358191	0.172 ± 0.173^a^	0.932 ± 0.282^d,e^	0.000 ± 0.000	0.000 ± 0.000	0.000
EP2	45336733-45336780	0.756 ± 1.309	1.801 ± 1.081	0.000 ± 0.000	0.218 ± 0.218	0.168
	45336822-45336870	0.766 ± 1.327	2.247 ± 1.320	0.128 ± 0.221	0.671 ± 0.131	0.143
	45336828-45336876	0.727 ± 1.259	2.151 ± 1.257	0.145 ± 0.252	0.892 ± 0.106	0.151
EP3	45336822-45336870	0.766 ± 1.327	2.247 ± 1.320	0.263 ± 0.263	0.000 ± 0.000	0.123
	45336828-45336876	0.727 ± 1.259	2.151 ± 1.257	0.395 ± 0.421	0.000 ± 0.000	0.145
EP4	34398855-34398913	1.650 ± 1.060 ^b^	0.833 ± 0.547	0.000 ± 0.000	0.000 ± 0.000	0.045
	34398899-34398948	2.423 ± 1.677 ^b^	1.681 ± 1.018	0.000 ± 0.000	0.000 ± 0.000	0.063
EP5	43316483-43316539	0.775 ± 0.930	2.116 ± 0.842	0.297 ± 0.515	0.685 ± 0.428	0.072
	43316494-43316553	0.786 ± 0.972	2.135 ± 0.914	0.207 ± 0.358	0.470 ± 0.392	0.057
	43316641-43316700	0.538 ± 0.683	0.979 ± 1.097	0.446 ± 0.509	0.871 ± 0.029	0.814
EP6	43482611-43482655	1.159 ± 1.335	2.887 ± 1.844	0.000 ± 0.000	0.000 ± 0.000	0.070
	43482725-43482778	1.624 ± 0.792^b,c^	2.405 ± 0.651^d,e^	0.000 ± 0.000	0.000 ± 0.000	0.001
EP7	43484105-43484160	1.505 ± 0.922^b,c^	1.695 ± 0.366^d,e^	0.000 ± 0.000	0.000 ± 0.000	0.001
	43484227-43484272	0.430 ± 0.656	1.384 ± 0.964^d^	0.000 ± 0.000	0.000 ± 0.000	0.092
EP8	23481701-23481760	0.215 ± 0.372	0.000 ± 0.000	0.000 ± 0.000	0.000 ± 0.000	0.525
EP9	30214808-30214863	0.000 ± 0.000	0.036 ± 0.062	0.000 ± 0.000	0.167 ± 0.167	0.165
EP10	33346983-33347035	1.157 ± 0.930	1.937 ± 0.338^d,e^	0.000 ± 0.000	0.000 ± 0.000	0.005
	33346985-33347037	1.170 ± 1.065	1.951 ± 0.301^d,e^	0.000 ± 0.000	0.000 ± 0.000	0.010
EP11	45254824-45254879	0.872 ± 0.896	2.002 ± 0.406	0.511 ± 0.642	1.212 ± 0.173	0.088
EP12	38919376-38919423	0.639 ± 1.106	1.762 ± 0.963	0.590 ± 0.700	1.393 ± 0.050	0.361
	38919458-38919517	0.814 ± 1.249	1.911 ± 0.983	0.471 ± 0.651	1.412 ± 0.073	0.317
	38919529-38919577	0.812 ± 0.948	1.556 ± 0.651	0.329 ± 0.570	1.122 ± 0.165	0.256

Data are presented as mean±standard deviation. Levels were compared using robust one-way analysis of variance (ANOVA), the post hoc Tamhane’s T2 test for multiple comparisons. In post hoc analysis, statistical significance (*P* < 0.05) is presented as follows: ^a^Trisomy 21 placenta versus normal placenta; ^b^Trisomy 21 placenta versus pregnant women blood; ^c^Trisomy 21 placenta versus non pregnant women blood; ^d^Normal placenta versus pregnant women blood; ^e^Normal placenta versus non pregnant women blood.

The CNVs of the 12 EPs is presented in Table 4. Six of 12 EPs (EP2, EP3, EP5, EP8, EP10, and EP11) were located within chromosomal regions with CNVs. Among the four EPs (EP1, EP6, EP7, and EP10) showing tissue-specific epigenetic characteristics between placenta and blood, EP10 was included within chromosomal regions with CNVs which can cause incorrect interpretation of results. Finally, EP6 and EP7 were selected as potentially fetal-specific EPs for the NIPT of fetal T21.

**Table 4:** Copy number variation of EPs

**EP**	**Position of EPs according to assembly**		**Copy number variation**
	**NCBI36/hg18**	**GRCh37/hg19**	**(Database of genomic variant)**
EP1	39279856-39280004	40357986-40358134	
EP2	44161178-44161323	45336750-45336895	Variation_7327 (Loss)
EP3	44161239-44161371	45336811-45336943	Variation_7327 (Loss)
EP4	33320735-33320829	34398865-34398959	
EP5	42189557-42189683	43316488-43316614	Variation_10602 (Loss)
EP6	42355712-42355815	43482643-43482746	
EP7	42357215-42357341	43484146-43484272	
EP8	22403649-22403792	23481778-23481921	Variation_30142 (Loss)
EP9	29136735-29136844	30214864-30214973	
EP10	32268843-32268943	33346972-33347072	Variation_4114 (Loss/Gain)
EP11	44079235-44079322	45254807-45254894	Variation_5162 (Loss),Variation_7327(Loss), Variation_73647 (Loss), Variation_79379 (Gain) Variation_90815 (Loss), Variation_90816 (Loss)
EP12	37841284-37841411	38919414-38919541	

### Confirmation of tiling oligonucleotide array data using bisulfite direct sequencing

**Figure 2:**
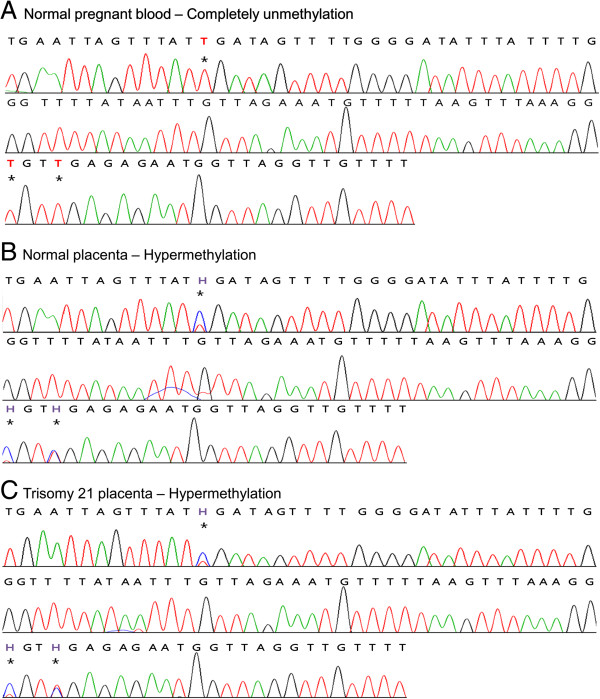
**Bisulfite direct sequencing of EP6.** The asterisk represents CpG sites in EP6. Red and blue peaks in sequences indicate T and C bases, respectively. Red (T) and violet (H) characters represent unmethylated cytosine and heterozygous cytosine, respectively, in CpG sites. CpG sites of EP6 were completely unmethlylated in normal pregnant blood **(A)** and were hypermethylated in both normal placenta **(B)** and trisomy 21 placenta **(C)**.

**Figure 3:**
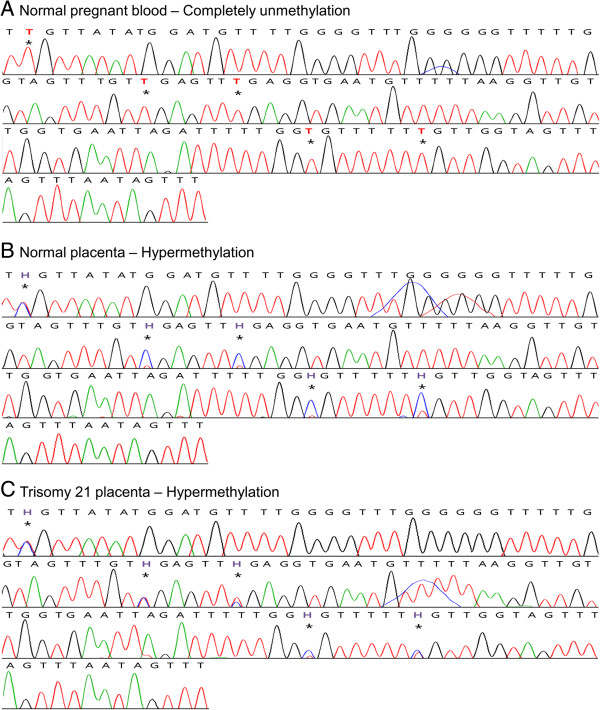
**Bisulfite direct sequencing of EP7.** The asterisk represents CpG sites in EP7. Red and blue peaks in sequences indicate T and C bases, respectively. Red (T) and violet (H) characters represent unmethylated cytosine and heterozygous cytosine, respectively, in CpG sites. CpG sites of EP7 were completely unmethlylated in normal pregnant blood **(A)** and were hypermethylated in both normal placenta **(B)** and trisomy 21 placenta **(C)**.

**Figure 4:**
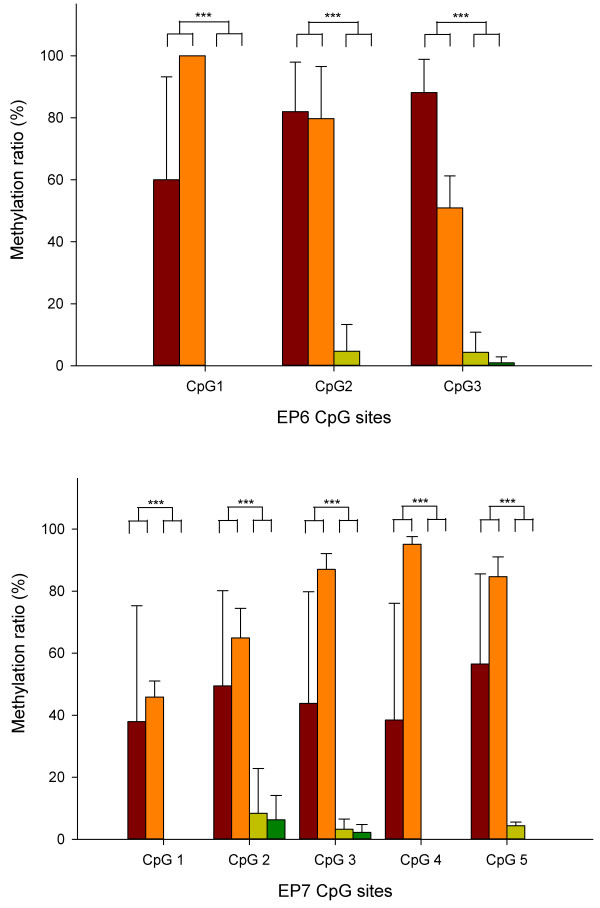
**Methylation ratio of each CpG site in EP6 and EP7 by bisulfite direct sequencing.** Data are presented as mean ± standard deviation. Red bars, Trisomy 21 placenta samples (n = 5); orange bars, normal placenta samples (n = 6); yellowish bars, pregnant normal whole blood (n = 8); green bars, non-pregnant normal whole blood (n = 4). *** *P* <0.001.

We confirmed that the CpG sites of EP6 and EP7 were hypermethylated in placenta compared with blood using bisulfite direct sequencing (Figures 2 and 3). Whole blood samples from non-pregnant euploid women (n = 4), whole blood samples from pregnant euploid women (n = 8), euploid fetal placenta samples (n = 6), and T21 fetal placenta samples (n = 5) were used. The CpG sites of EP6 and EP7 showed a hypermethylated pattern in placenta compared with blood, regardless of the presence or absence of disease and pregnancy (Figure 4). The methylation ratio in each EP6 and EP7 CpG site was significantly increased in placental samples compared with blood (*P* < 0.001 for all CpG sites). However, methylation ratio between T21 and normal placental samples was not different (*P* > 0.05 for all CpG sites).

### Confirmation of tiling oligonucleotide array data using qPCR

We investigated the tissue-specific methylation levels of EP in whole blood samples from non-pregnant euploid women (n = 6), whole blood samples from pregnant euploid women (n = 16), euploid fetal placenta samples (n = 14), and T21 fetal placenta samples (n = 11) using qPCR (Table 5). The ΔCt of EP6 and EP7 in whole blood samples from non-pregnant women and pregnant women carrying euploid fetuses showed a negative value and presented a hypomethylated pattern of EPs, whereas the ΔCt of the EP6 and EP7 in euploid and T21 placenta samples showed a positive value and presented a hypermethylated pattern of EPs. The ΔCt of the EP6 and EP7 were significantly higher in T21 and euploid placenta samples than in whole blood samples from pregnant women carrying euploid fetuses and non-pregnant women (*P* < 0.001 for all). In addition, we selected EP9 as a negative marker based on array data and investigated its methylation levels (Table 5). The ΔCt of EP9 showed a negative value and presented a hypomethylated pattern in all cases, regardless of the presence or absence of disease and pregnancy. There was no statistically significant difference.

**Table 5:** Ct values of EPs by qPCR

**EP**		**Placenta**		**Blood**		***P *** **value**	**Ct**_**MBD **_**of cff-DNA (n = 20)**	***P *** **value**
		**Trisomy 21 (n = 11)**	**Normal (n = 14)**	**Pregnant (n = 16)**	**Non-pregnant (n = 6)**			
EP6								
	Ct_input_	22.3±0.4	23.4±1.5	23.1±2.3	22.3±0.1	0.364	29.4 ±1.6^†^	0.021
	Ct_MBD_	20.9±0.9^a,b^	20.9±1.0^c,d^	29.3±1.1	29.1±2.3	<0.001		
	ΔCt	1.5±0.9^a,b^	2.5±0.9^c,d^	−6.2±2.2	−6.7±2.4	<0.001		
EP7								
	Ct_input_	22.8±0.9	24.8±2.5	24.0±2.7	21.8±0.6	0.143	29.5±1.5^#^	
	Ct_MBD_	20.6±0.6^a,b^	21.4±0.8^c,d^	29.3±0.9	27.4±1.7	<0.001		
	ΔCt	2.2±1.1^a,b^	3.4±2.2^c,d^	−5.3±2.9	−5.6±1.2	<0.001		
EP9								
	Ct_input_	20.7±0.2	21.9±0.6	21.2±0.6	22.9±0.1	0.243	34.8±4.6	
	Ct_MBD_	28.8±0.6	28.9±1.2	27.0±1.7	28.6±2.0	0.469		
	ΔCt	−8.1±0.4	−7.1±0.8	−5.8±2.1	−5.6±2.1	0.421		

Data are presented as mean±standard deviation. Ct_input_ denotes the threshold cycle of the input portion without methylated DNA enrichment. Ct_MBD_ denotes the threshold cycle of the methylated DNA enriched portion. The delta (Δ) threshold cycle (Ct) value was calculated as ΔCt = Ct_input_ - Ct_MBD_. The values were compared using robust one-way analysis of variance (ANOVA), the post hoc Tamhane’s T2 test for multiple comparisons. In post hoc analysis, statistical significance (*P* < 0.05) is presented as follows: ^a^Trisomy 21 placenta versus pregnant women blood; ^b^Trisomy 21 placenta versus non pregnant women blood; ^c^Normal placenta versus pregnant women blood; ^d^Normal placenta versus non pregnant women blood; ^†^EP6 versus EP9; ^#^EP7 versus EP9.

### Noninvasive detection of selected EPs in maternal plasma

We investigated possibility for noninvasive detection of selected EPs in maternal plasma using qPCR. The maternal plasma samples from 20 pregnant euploid women were used. EP6, EP7, and EP9 levels were measured in all samples without failure of MBD capture. Ct value of EP6, EP7, and EP9 were 29.4 ± 1.6, 29.5 ± 1.5, and 34.8 ± 4.6, respectively. There was no statistically significant difference between Ct values of EP6 and EP7 (*P* = 0.902). However, Ct value of EP9 was significantly higher than those of EP6 and EP7 (*P* < 0.001, Table 5).

## Discussion

In this study, we validated that EP6 and EP7 have a hypermethylated pattern in placenta compared with blood, regardless of the presence or absence of disease and pregnancy and are detectable in maternal plasma. Our results demonstrate that the other ten EPs showed methylation discrepancies that are opposite previous microarray data, which were obtained using whole blood from non-pregnant women and euploid placental samples or were located in CVN regions which can result in dosage imbalances. Therefore, we suggest that EP6 and EP7 have potential as effective biomarkers for the non-invasive detection of fetal T21, and that disease-specific epigenetic characteristic should be considered for development of fetal-specific EPs for the NIPT of fetal T21.

In general, 5′-methylcytosine accounts for approximately 1% of total DNA bases in humans, and therefore potentially affects 70-80% of all CpG dinucleotides in the genome [21]. Moreover, DNA methylation patterns are dynamic and vary during development and across the genome. DNA methylation has been a topic of considerable interest in development of biomarkers for prognosis and diagnosis of various human diseases including a variety of tumors, Alzheimer’s disease, T21, and others [22-25], because DNA methylation has disease-specific epigenetic characteristics that vary in the same tissue according to the presence or absence of disease. In a previous study investigating the alteration of DNA methylation depending on T21 disease status, DNA methylation in total peripheral blood leukocytes and T-lymphocytes from patients with T21 and normal controls has been profiled and found gene-specific abnormalities of CpG methylation in patients with T21 [22]. Therefore, to understand disease-specific epigenetic characteristics it is important to develop effective EPs for prognosis and diagnosis of disease.

Recently, the use of epigenetic differences between maternal whole blood and placental DNA has become key area of interest for the development of the NIPT of fetal T21. The quantification of fetal DNA by methylation-based DNA discrimination has been used for the NIPT of fetal T21 and promising results have been reported [16-18]. Such fetal-specific EPs on chromosome 21 are applied for the detection of fetal T21 by direct comparison with a placenta-derived DNA methylation marker on a reference chromosome. Theoretically, the methylation ratio of fetal-specific EPs may present equal signal intensity for unaffected fetuses and an increased signal intensity of chromosome 21 for T21 fetuses, because T21 is caused by an extra copy of all or part of chromosome 21. To do that, fetal-specific EPs on chromosome 21 should be hypermethylated in the placenta and unmethylated in the blood, regardless of the presence or absence of pregnancy and T21. However, until now, fetal-specific EPs for the NIPT of fetal T21 have been identified based on the tissue-specific epigenetic characteristics between placenta and maternal blood. The EPs analyzed in this study were also markers identified by the only tissue-specific epigenetic characteristics between whole blood from non-pregnant women (n = 5) and euploid placenta samples (first trimester: n = 3, third trimester: n = 2) in a previous study [19]. Epigenetic alteration of these EPs according to the presence or absence of pregnancy and disease has not been validated. Nevertheless, the new platform using the methylation ratio of the EPs has been exhibited excellent clinical performance (both the sensitivity and specificity were 100%) [18,26]. However, Tong *et al.* reported a low diagnostic accuracy (a sensitivity of 33% and a specificity of 73%), in contrast to the perfect classification reported previously [27]. In this study, we found that most EPs, except EP6 and EP7, showed discrepant methylation patterns among the tested samples according to the presence or absence of pregnancy and T21. Furthermore, only one T21 case seemed methylation pattern similar to normal cases. This indicates that most EPs identified in a previous study are likely to lead to inaccurate results in non-invasive detection of fetal T21. Therefore, the reproducibility and precision in a realistic clinical setting of using fetal-specific EPs that do not have disease-specific epigenetic characteristics is controversial. More research is needed to confirm utility of fetal-specific EPs for the NIPT of fetal T21. This study was also limited by its small sample size and the inclusion of only Korean subjects; therefore, a larger-scale study within different ethnic populations is needed. Moreover, EPs based on disease-specific epigenetic characteristics need to be identified for the NIPT of fetal T21.

## Conclusion

In conclusion, we confirmed that EP6 and EP7 had potential for use as fetal-specific EPs for the NIPT of fetal T21, due to their consistent disease-specific epigenetic characteristics. Therefore, we suggest that consideration of disease-specific epigenetic characteristics of fetal EPs should certainly take precedence in the development of effective and reliable EPs for the NIPT of fetal T21. Eventually, the identification of effective fetal-specific EPs through the consideration of epigenetic characteristics in disease and tissue may reduce the complexity and cost of these test by excluding the use of inappropriate biomarkers that produce imprecise results in the NIPT of fetal T21.

## Abbreviations

T21: Trisomy 21; NIPT: Non-invasive prenatal testing; cff-DNA: Cell-free fetal DNA; EPs: Epigenetic markers; DMRs: Differentially methylated regions; CVS: Chorionic villus sampling; MBD: Methyl-CpG binding domain-based protein; CNVs: Copy number variations.

## Competing interests

The authors have declared that no competing interests exist.

## Authors’ contributions

JHL and DEL participated in study design, execution, analysis, and manuscript drafting. HKA, YJH, and MYK collected samples and participated in interpretation of data and critical discussion. DJK participated in experimental execution and analysis. SYP participated in study design, interpretation of data, and manuscript revision. HMR participated in study design, sample collection, manuscript editing and critical discussion. All authors read and approved the final manuscript.

## Pre-publication history

The pre-publication history for this paper can be accessed here:

http://www.biomedcentral.com/1755-8794/7/1/prepub
